# Cross-cultural adaptation of the Dysphonia Screening Tool (DST-Br) for European Portuguese (EP)

**DOI:** 10.1590/2317-1782/20232023080en

**Published:** 2023-12-18

**Authors:** Cláudia Correia, Vanessa Veis Ribeiro, Priscila Oliveira Costa Silva, Mara Behlau

**Affiliations:** 1 Centro de Estudos da Voz - CEV - São Paulo (SP), Brasil.; 2 Curso de Fonoaudiologia, Faculdade de Ceilândia, Universidade de Brasília - UnB - Brasília (DF), Brasil.; 3 Departamento de Fonoaudiologia, Universidade Federal da Paraíba - UFPB - João Pessoa (PB), Brasil.

**Keywords:** Self-Assessment, Dysphonia, Speech Language and Hearing Sciences, Surveys and Questionnaires, Triage, Voice

## Abstract

**Purpose:**

To perform a cross-cultural adaptation of the Brazilian Dysphonia Screening Tool (DST-Br) for European Portuguese (EP).

**Methods:**

The cross-cultural adaptation of the DST-Br for EP was carried out in four stages: translation, back-translation, expert committee review, and pre-testing. The pre-testing involved 30 dysphonic individuals (24 women and 6 men) aged between 18 and 87 years old.

**Results:**

An additional statement was required in the EP version of the instrument. Disagreement in the back-translation of the title was resolved through an expert committee review. One item presented discrepancies in the translation and back-translation, with the final version determined through an expert committee review. One item and the answer key reached a consensus in all stages. During pre-testing, all items received 100% "yes" or "no" responses, and none were marked as "not applicable".

**Conclusion:**

The cross-cultural adaptation of DST-Br for use in EP was successfully carried out. The European Portuguese version of the instrument was named the Instrumento de Rastreio para a Disfonia em português europeu (IRD-PT) / Dysphonia Screening Tool in European Portuguese.

## INTRODUCTION

Dysphonia affects approximately one-third of the global population at some point in their lives. It is characterized by alterations in vocal quality, pitch, loudness, or vocal effort and can significantly impact individuals' communication and quality of life^(^1,2^)^.

Usually, the measurement of individuals' self-perception regarding symptoms, discomfort, and the consequences of dysphonia on their quality of life is assessed using vocal self-assessment questionnaires^(^3^)^. These instruments enable the assessment of the person’s perception, which cannot be obtained with other forms of evaluation^(^4^)^.

Many of these questionnaires have been developed aiming to assess the person’s self-perception regarding their clinical state and the dysphonia impact on their lives^(^5^)^, including the Voice Handicap Index with 10 items (VHI-10) translated and validated to European Portuguese (EP)^(^1^)^ and the Voice Symptom Scale (VoiSS) translated and adapted to EP^(^6^)^. These instruments have high accuracy and significant scientific and clinical value. They also present cutoff points that aid in distinguishing individuals with or without dysphonia. However, it's noteworthy that they were not originally developed for screening voice disorders.

To address the need for a screening tool for dysphonia, in 2020, the Dysphonia Screening Tool was developed in Brazilian Portuguese (DST-Br)^(^7^)^. The DST-Br is a straightforward and highly accurate instrument with two items that are easily discerned. These items were extracted from two classic self-assessment vocal instruments, the VHI and the VoiSS. To identify individuals with a high likelihood of having dysphonia and perform the proper referring, the DST-Br employs only the items "My voice is hoarse" ("*Minha voz é rouca*" in Brazilian Portuguese) and "I feel as though I have to strain to produce voice" ("*Sinto que tenho que fazer força para a minha voz sair*" in Brazilian Portuguese)^(^7^)^.

However, there is no version of this instrument available in EP nor is there any other screening or assessment tool for vocal disorders in this language. Hence, there is a need to cross-culturally adapt the DST-Br for EP to assist clinicians in identifying the risk of dysphonia in the Portuguese population in an easy, quick, and accurate manner.

Therefore, this study aimed to perform a cross-cultural adaptation of the Brazilian Dysphonia Screening Tool (DST-Br) for EP.

## METHODS

This study was approved by the Research Ethics Committee of *Universidade de Taubaté*, University of Taubaté (protocol number 5.162.826). All participants received information regarding the study and signed the Informed Consent Form using the Google Forms platform. The research followed the CNS 466/12 resolution guidelines.

The DST-Br translation and adaptation to EP were conducted in accordance with the criteria proposed by the *COnsensus-based Standards for the selection of health Measurement Instruments* (COSMIN)^(^8^)^. The DST-Br has two items: (1st) "I feel as though I have to strain to produce voice" *("Sinto que tenho que fazer força para a minha voz sair"* in Brazilian Portuguese) from VHI-10 and (2nd) "My voice is hoarse" *("Minha voz é rouca"* in Brazilian Portuguese) from VoiSS. Although both items have their EP versions in the respective original instruments from which they were derived, the decision was made to translate the entire instrument, including its name, items, and response key.

The stages of translation, back-translation, and expert committee review are specified as follows:

1. Translation: The translations were performed by two translators, one was a speech-language pathologist, and the other was not. Both were women native speakers of EP and fluent in Brazilian Portuguese (BP). Both translators independently translated the information.
2. Consensus: The authors reached a consensus between the two translations regarding the title, the items, and the response key.
3. Back-translation: The consensus version underwent back-translation into BP by two translators (one was a speech-language pathologist, and the other was not); both were fluent in EP. Their purpose was to determine if there were any significant alterations in the original content.
4. Expert Committee Review: The title, the two items, and the response key underwent an analysis by an expert committee of speech-language pathologists who did not participate in the previous stages. The committee included a methodologist, a Brazilian speech-language pathologist, and three Portuguese speech-language pathologists. The expert committee analysis considered the semantic, conceptual, idiomatic, experiential, cultural, and operational equivalence^(^9^)^ discrepancies observed during the translation and back-translation processes.
5. Pre-testing: The instrument's final version underwent pre-testing, which involved its administration to native Portuguese-speaking individuals with dysphonia who were living in Portugal. These participants were recruited through invitations sent to a Portuguese speech-language pathologist. The invitation included the inclusion criteria and the link for participation. The Portuguese speech-language pathologist was asked to extend the invitation to their patients who met the research eligibility criteria. Inclusion criteria were: be +18 years old, native to Portugal, and with a medical diagnosis of dysphonia. Exclusion criteria were the presence of neurological, cognitive, and/or psychiatric issues that would make their understanding of the research items difficult. Participants who accepted signed an online Informed Consent Form. Next, they received a link with two screens. The first screen included five questions such as name, age, sex, profession, and medical diagnosis. The second screen presented the instrument with its two items. A "not applicable" option was added to the answer key; participants were instructed to select this option for items that were not appropriate for their culture. The final sample consisted of 30 dysphonic individuals aged between 18 and 87 years old (mean age of 50 years and seven months, SD = 18.55); there were 24 women (80%) and six men (20%).

All stages were conducted online. Google Forms was used for data collection for the pre-testing.

## DATA ANALYSIS

The data were analyzed descriptively and inferentially using SPSS 25.0 software. The significance level was set at 5% for inferential analyses.

In the descriptive analysis of quantitative variables, measures of central tendency (mean and median), variability (standard deviation), and position (minimum, maximum, first and third quartiles) were calculated. For the descriptive analysis of qualitative variables, absolute frequency, and relative frequency in percentages were computed.

The comparison of the proportion of two categories of a nominal qualitative variable was carried out using a One-Sample Binomial Test, with a reference proportion of 0.5. When comparing multiple categories of a nominal qualitative variable, the reference proportion was set as the proportion of the category with the highest frequency.

## RESULTS

The proposed statement for the DST-PT was based on the items of both instruments (VHI-10^(^1^)^ and VoiSS^(^6^)^) used to create the original version of the DST-Br. The DST-Br does not have an introduction before presenting the two items. Chart 1 shows the statement outcomes of the DST-PT.

**Chart 1:** Statement outcomes for the DST-PT

**Original Statement in EP**		**Statement Proposal**		**Chosen statement by the authors**			
		**DST-PT**					
VHI-10	*"Estas são afirmações usadas para descrever o efeito da voz na qualidade de vida. Escolha a opção que indica com que frequência teve a mesma experiência. (Nunca=0 pontos; Quase nunca= 1 ponto; às vezes= 2 pontos; Quase sempre= 3 pontos; Sempre=4 pontos).*	Option 1	***Responda aos dois itens abaixo, considerando a sua voz atualmente.***	x	x	x	x
		Option 2	*Responda sim ou não às seguintes questões sobre a sua voz*				
VoiSS	*"Desenhe um círculo à volta de uma resposta em cada item. Não deixe itens em branco."*	Option 3	*Responda às seguintes questões.*				
		Option 4	*Considerando a sua voz, responda às duas questões abaixo.*				

Chart 2 presents the translation, back-translation, and expert committee review results.

**Chart 2:** Description of the translation, back-translation, and expert committee review

**Statement: *Responda aos dois itens abaixo, considerando a sua voz atualmente*.**				
**Original Version**	**Translation**	**Consensus**	**Back-translation**	**Expert Committee**
		**(European Portuguese)**		
**Title**				
*Instrumento de rastreio da disfonia*	*- Instrumento de rastreio para a disfonia*	***Instrumento de rastreio para a disfonia***	*- Questionário para triagem de disfonia*	*Instrumento de rastreio para a disfonia*
	*- Instrumento de rastreio de disfonia*		- Instrumento de rastreio para a disfonia	
**Answer key for each item**				
sim	- sim	**sim**	sim	sim
	- sim		sim	
não	- não	**não**	não	não
	- não		não	
**Items**				
1. Sinto que tenho que fazer força para a minha voz sair	- Sinto que tenho de fazer força para a minha voz sair	**Sinto que tenho de fazer força para a minha voz sair**	- Sinto que faço força para a minha voz sair	Sinto que faço força para a minha voz sair
	- Sinto necessidade de fazer força para a minha voz sair		- Sinto que tenho que fazer força para a minha voz sair	
2. Minha voz é rouca	- A minha voz é rouca	**A minha voz é rouca**	- A minha voz é rouca	A minha voz é rouca
	- A minha voz é rouca		- A minha voz é rouca	

The participants had the following medical diagnoses: functional dysphonia (n=11; 36.67%), organic-functional dysphonia (n=9; 30%), and organic dysphonia (n=10; 33.33%). Table 1 indicates that no participant responded "not applicable" for any of the instrument items. For the item "I feel as though I have to strain to produce voice” 46.67% of participants answered "no", while 53.33% answered "yes". As for the item, “My voice is hoarse," 20% of participants answered "no", and 80% answered "yes".

**Table 1:** Proportion of responses in the instrument items

Variables and categories	n	%	p-value
*Sinto que tenho de fazer força para a minha voz sair /* I feel that I strain to produce my voice			
*Não/Sim* No/Yes	30	100.00	1.000
*Não aplicável* Not applicable	0	0.00	
*A minha voz é rouca* / My voice is hoarse			
*Não/Sim* No/Yes	30	100.00	1.000
*Não aplicável* / Not applicable	0	0.00	

One-Sample Binomial Test

**Caption:** n=absolute frequency; %=relative frequency

## DISCUSSION

Cross-cultural adaptation aims at modifying the items of an instrument to make it applicable within a different population considering language and culture. It addresses potential sociocultural disparities between cultures and languages instead of being a literal translation of the original instrument, thereby enabling its use in a format suitable for individuals within the target culture^(^10^)^. Only after cross-cultural adaptation can the instrument be used in a secondary language and culture.

The DST-Br translation into EP did not present any discrepancies in the content. There was no need for adjustment in the answer key. Regarding the title, there was agreement in the translation; however, in the back-translation, two possibilities emerged: *“questionário para a triagem”*, in English "questionnaire for screening", or *“instrumento de rastreio”*, in English "screening tool." The term “*instrumento*”, i.e., “tool”, was kept due to the conceptualization and the DST usage format. The term "questionnaire" is used for an instrument of data collection, with various questions, and it is a more limited concept than "instrument." In contrast, an “instrument” refers to what will be used in the study's development for data acquisition^(^11^)^ and may have specific forms of analysis that support decision-making.

The DST-Br seeks to obtain data and also to provide a formula for the clinician's decision-making based on the patient's responses. The DST is understood to be more than just a questionnaire for obtaining isolated pieces of information.

The concept of *"rastreio"*, in English "screening", was chosen over *"triagem"* in English "triage" once *"rastreio"* involves the simple, cost-effective, and rapid detection of a potential disease or condition, followed by referral for diagnosis confirmation and treatment. *"Triagem"*, on the other hand, identifies individuals affected by a disease or disorder within a population to address them with more complete diagnostic procedures^(^7^)^. Therefore, the concept of *"rastreio”* was chosen by the expert committee as it aligns with the DST-Br purpose, which is to identify individuals with voice disorders in a simple, cost-effective, and quick manner and refer them for diagnosis confirmation and treatment.

Regarding the items, the premise of simplification was employed during the translation and back-translation consensus stage. Syntactic simplification is a psycholinguistics practice supported by evidence that certain grammatical features can create greater or lesser difficulties for text comprehension^(^12^)^. Hence, simplification involves various strategies aimed at reducing the text's structural complexity without changing the content of the original information, i.e., its meaning. This simplification allows for a broader reach to individuals of diverse socioeconomic and cultural backgrounds. Thus, the first item showed discrepancies in translation, with the translation being defined as: "*Sinto que tenho de fazer força para a minha voz sair*" ("I feel as though I have to strain to produce voice "). There was also a disagreement in the back-translation. Hence, during the expert committee's review, it was modified to "*Sinto que faço força para a minha voz sair*" ("I feel that I strain to produce my voice"), making the item simpler and easier to understand. The second item did not undergo any changes, it maintained the same version in all stages. For the sentence to become clear in EP, since the first stage of the translation and adaptation from the BP, the article “a” was added in the beginning of the sentence.

The original instrument does not have a formal statement to introduce its questions. However, there was a need to include an introduction to the instrument, especially due to the online application to clarify its purpose and provide filling instructions for the participants. Considering the two instruments (VHI-10; and VoiSS) that served as guidance for the creation of the DST, the introduction statement was based on the EP versions of the VHI-10 and VoiSS. Four introduction options were created, from which the authors selected the following: *“Responda aos dois itens abaixo, considerando a sua voz atualmente*” (“Answer the two items below, considering your voice at the moment”).

The "not applicable" option was not selected by the participants during the pre-testing. This stage's outcomes are considered excellent, as all 30 participants responded either affirmatively or negatively to the two items under study. Hence, they understood the items and responded within the response key of the instrument itself. This demonstrates that the instrument adaptation for EP achieved a final version, which is easy to understand by the Portuguese population.

Cross-cultural adaptation is the first step for the validation of instruments once it is essential for the following stages^(^10^)^. With the conclusion of the cross-cultural adaptation process for EP, it is now necessary to begin the DST-PT validation study. This validation study aims to demonstrate the psychometric properties of validity, reliability, and accuracy of the Portuguese version, enabling its reliable use in research and clinical practice^(^13^)^.

## CONCLUSION

The cross-cultural translation and adaptation of DST-Br for use in the European Portuguese population was successfully carried out. The European Portuguese version of the instrument was named the *Instrumento de Rastreio para a Disfonia em português europeu* (IRD-PT) / Dysphonia Screening Tool in European Portuguese (see Appendix 1).

## Appendix 1 INSTRUMENTO DE RASTREIO PARA A DISFONIA - IRD^PT^ (in European Portuguese)

Responda aos dois itens abaixo, considerando a sua voz atualmente.

**Table:**

1) Sinto que tenho de fazer força para a minha voz sair	( ) Sim ( ) Não
2) A minha voz é rouca	( ) Sim ( ) Não
