# Supplementary material for: Cross-cultural adaptation of the Dysphonia Screening Tool (DST-Br) for European Portuguese (EP)
Source: Codas. 2023 Dec 18;36(2):e20230080. doi: 10.1590/2317-1782/20232023080en (PMC10750892; doi:10.1590/2317-1782/20232023080en)
Supplement: Translation

# Translation: Adaptação transcultural do Instrumento de Rastreio para a Disfonia (IRD-Br) para o Português Europeu (PE)

**DOI:** 10.1590/2317-1782/20232023080pt

**Authors:** Cláudia Correia, Vanessa Veis Ribeiro, Priscila Oliveira Costa Silva, Mara Behlau

**Affiliations:** 1 Centro de Estudos da Voz - CEV - São Paulo (SP), Brasil.; 2 Curso de Fonoaudiologia, Faculdade de Ceilândia, Universidade de Brasília - UnB - Brasília (DF), Brasil.; 3 Departamento de Fonoaudiologia, Universidade Federal da Paraíba - UFPB - João Pessoa (PB), Brasil.

**Keywords:** Autoteste, Disfonia, Fonoaudiologia, Inquéritos e Questionários, Triagem, Voz

## Abstract

**Objetivo:**

Realizar a adaptação transcultural do Instrumento de Rastreio para a Disfonia (IRD-Br) para o Português Europeu (PE).

**Métodos:**

Foi realizada a adaptação transcultural do IRD-Br para o PE de acordo com as seguintes etapas: tradução, retrotradução, análise de um comitê de especialistas e pré-teste. Na etapa de pré-teste, participaram 30 indivíduos disfônicos com idades entre os 18 e 87 anos, sendo 24 do sexo feminino e 6 do sexo masculino.

**Resultados:**

Foi necessária a inserção de um enunciado na versão em PE do instrumento. Houve divergência na retrotradução do título, sendo resolvida na análise do comitê de especialistas. Um item apresentou divergência na tradução e na retrotradução, sendo definida a versão final na análise do comitê de especialistas. Um item e a chave de resposta apresentaram consenso em todas as etapas. No pré-teste, todos os itens receberam 100% de respostas sim ou não, e nenhum recebeu resposta não aplicável.

**Conclusão:**

A adaptação do IRD-Br para o PE foi bem sucedida. A versão para o português europeu do instrumento foi denominada de Instrumento de Rastreio para a Disfonia em português europeu - IRD-PT.

## INTRODUÇÃO

A disfonia afeta cerca de um terço da população mundial em algum momento da sua vida. Ela é caracterizada por uma mudança na qualidade, *pitch*, *loudness* ou esforço vocal, e pode impactar na comunicação e na qualidade de vida dos indivíduos^(1,2)^.

A mensuração da percepção dos próprios indivíduos acerca dos sintomas, desconfortos e das consequências da disfonia na sua qualidade de vida é geralmente realizada por questionários de autoavaliação vocal^(3)^. Esses instrumentos permitem mensurar a percepção do sujeito, o que não pode ser obtido por outro tipo de avaliação^(4)^.

Muitos questionários foram criados com o objetivo de avaliar a autopercepção do sujeito acerca do quadro clínico e do impacto da disfonia^(5)^, entre eles: Índice de Desvantagem Vocal reduzido - IDV-10 (Voice Handicap Index-10) traduzido e validado para o Português Europeu (PE)^(1)^ e a Escala de Sintomas Vocais (ESV) traduzida e adaptada para o PE - VoiSS (Escala de Sintomas de Voz)^(6)^. Esses são instrumentos com elevada acurácia e alto valor científico e clínico. Esses instrumentos possuem pontos de corte que auxiliam na diferenciação de indivíduos com ou sem disfonia, no entanto não foram desenvolvidos para esse objetivo, o de rastrear distúrbios da voz.

A fim de suprir a necessidade de ter um instrumento para rastrear a disfonia, em 2020 foi elaborado o Instrumento de Rastreio para a Disfonia (IRD-Br)^(7)^ em português brasileiro, nomeado como *Brazilian Dysphonia Screening Tool* (Br-DST) em inglês. O IRD-Br é um instrumento simples e de boa acurácia, composto por dois itens que são facilmente percebidos pelos indivíduos, e que foram extraídos de dois instrumentos clássicos de autoavaliação vocal, o IDV e a ESV. O IRD-Br usa apenas os itens "Minha voz é rouca?" e "Sinto que tenho que fazer força para a minha voz sair?" para identificar indivíduos com alta probabilidade de apresentar disfonia, e encaminhá-los para uma avaliação multidimensional da voz confirmatória^(7)^.

Porém, não há uma versão do instrumento disponível em PE, bem como, de nenhum outro instrumento de triagem ou rastreio de distúrbios vocais. Dessa forma, vê-se a necessidade de adaptar transculturalmente o IRD-Br para o PE para auxiliar o clínico na identificação do risco de disfonia na população portuguesa de uma forma fácil, rápida e precisa.

Assim, o objetivo do estudo foi realizar a adaptação transcultural do IRD-Br para o PE.

## MÉTODOS

A presente pesquisa foi aprovada pelo Comitê de Ética em Pesquisa da Universidade de Taubaté (parecer número 5.162.826). Todos os sujeitos foram esclarecidos sobre a pesquisa e assinalaram o aceite voluntário de participação na pesquisa na plataforma google forms. A pesquisa seguiu as normas de Resolução CNS 466/12.

A tradução e adaptação do IRD-BR para PE foi realizada de acordo com os critérios propostos pelo *COnsensus-based Standards for the selection of health Measurement Instruments* (COSMIN)^(8)^. O IRD-Br contém dois itens: (1º) "Eu sinto que tenho que fazer força para a minha voz sair" do IDV-10 e (2º) "A minha voz é rouca?" da ESV. Apesar de ambos os itens terem a sua versão em PE nos respectivos instrumentos originais que o compuseram, optou-se por traduzir o instrumento na totalidade (nome, itens e chave de resposta).

As etapas de tradução, retrotradução e análise do comitê estão especificadas abaixo:

1. Tradução: Foram realizadas por dois tradutores, um terapeuta da Fala e um não terapeuta da fala, nativos do PE e fluentes em Português brasileiro (PB). Ambos traduziram as informações de forma independente.
2. Consenso: Os autores realizaram um consenso entre as duas traduções do título, dos itens e da chave de resposta.
3. Retrotradução: A versão de consenso foi submetida à retrotradução para o PB, por dois tradutores (um fonoaudiólogo e outro não fonoaudiólogo) brasileiros fluentes em PE, com o objetivo de perceber se houve alguma alteração relevante no conteúdo original.
4. Análise do comitê: O título, os dois itens e a chave de respostas passaram por uma análise de um comitê de Fonoaudiólogos/Terapeutas da Fala não participantes nas etapas anteriores. O comitê foi constituído por um metodologista, um fonoaudiólogo e três Terapeutas da Fala. A análise feita pelo comitê de especialistas levou em conta a equivalência semântica, conceitual, idiomática, experiencial, cultural e operacional^(9)^ nas divergências encontradas na tradução e retrotradução.
5. Pré-teste: A versão final do instrumento passou pela etapa de pré-teste com a aplicação em indivíduos disfônicos portugueses falantes nativos do PE. Os participantes do pré-teste foram recrutados através de convite para participar da pesquisa enviados a Terapeutas da Fala (Fonoaudiólogos) em Portugal. Nesse convite constavam os critérios de seleção e o link para participação. Os Terapeutas de Fala foram convidados a repassar o convite aos seus pacientes que preenchiam os critérios de elegibilidade da pesquisa. Os critérios de inclusão foram: idade superior a 18 anos, ser nativo de Portugal e ter diagnóstico médico otorrinolaringológico de disfonia. Foram excluídos indivíduos com problemas neurológicos, cognitivos e/ou psiquiátricos que inviabilizassem a compreensão dos instrumentos de pesquisa. Os participantes que aceitaram o Termo de Consentimento Livre e Esclarecido (TCLE) no formulário on-line, foram encaminhados para um link que era composto por duas telas. Na primeira, responderam cinco questões que envolviam: nome, idade, sexo, profissão e diagnóstico médico otorrinolaringológico. A segunda tela continha o instrumento, com o enunciado e os dois itens de pesquisa. Foi adicionada a opção "não aplicável" à chave de resposta. Os participantes foram orientados a selecionar essa opção para os itens que estivessem inadequados para a sua cultura. A amostra final foi constituída por 30 indivíduos disfônicos com idades entre 18 e 87 anos, média de 50 anos e sete meses (DP: 18,55), sendo 24 do sexo feminino (80%) e seis do sexo masculino (20%).

Todas as etapas foram realizadas de forma online. Para o pré-teste foi utilizada a plataforma Google Forms.

## ANÁLISE DE DADOS

Os dados foram analisados de forma descritiva e inferencial utilizando-se o software SPSS 25.0. Foi considerado um nível de significância de 5% para as análises inferenciais.

Na análise descritiva das variáveis quantitativas foram calculadas as medidas de tendência central (média e mediana), variabilidade (desvio-padrão) e posição (mínimo, máximo, primeiro e terceiro quartis). Na análise descritiva das variáveis qualitativas foram calculadas a frequência absoluta e a frequência relativa percentual.

A comparação da proporção de duas categorias de uma variável qualitativa nominal foi realizada por meio do Teste Binominal de uma amostra, adotando-se a proporção de referência de 0,5. Para a comparação de múltiplas categorias de uma variável qualitativa nominal utilizou-se como referência a proporção da categoria de maior proporção.

## RESULTADOS

A proposta de enunciado para o IRD-PT teve como base os enunciados dos dois instrumentos (IDV-10^(1)^, VoiSS^(6)^) utilizados para a criação da versão original do IRD-Br. O IRD-Br não possui enunciado formal para introduzir os dois itens que compõem o instrumento. O Quadro 1 apresenta os resultados obtidos para o enunciado do IRD-PT.

**Quadro 1:** Resultados obtidos para o enunciado do IRD-PT

**Enunciados originais**		**Proposta de enunciado**		**Enunciado escolhido pelos autores**			
		**IRD- PT**					
IDV-10	*"Estas são afirmações usadas para descrever o efeito da voz na qualidade de vida. Escolha a opção que indica com que frequência teve a mesma experiência. (Nunca=0 pontos; Quase nunca= 1 ponto; às vezes= 2 pontos; Quase sempre= 3 pontos; Sempre=4 pontos).*	Opção 1	**Responda aos dois itens abaixo, considerando a sua voz atualmente.**	x	x	x	x
		Opção 2	Responda sim ou não às seguintes questões sobre a sua voz				
VoiSS	*"Desenhe um círculo à volta de uma resposta em cada item. Não deixe itens em branco."*	Opção 3	Responda às seguintes questões.				
		Opção 4	Considerando a sua voz, responda às duas questões abaixo.				

Os resultados obtidos na fase de tradução, retrotradução e análise do comitê estão especificadas no Quadro 2.

**Quadro 2:** Descrição das etapas de tradução, retrotradução e consenso

Enunciado: Responda aos dois itens abaixo, considerando a sua voz atualmente.				
Versão original	Tradução	Consenso	Retrotradução	Comitê de especialista
		(Português Europeu)		
**Título**				
Instrumento de rastreio da disfonia	- Instrumento de rastreio para a disfonia	**Instrumento de rastreio para a disfonia**	- Questionário para triagem de disfonia	Instrumento de rastreio para a disfonia
	- Instrumento de rastreio de disfonia		- Instrumento de rastreio para a disfonia	
**Opções de resposta para cada item**				
sim	- sim	**sim**	sim	sim
	- sim		sim	
não	- não	**não**	não	não
	- não		não	
**Itens**				
1. Sinto que tenho que fazer força para a minha voz sair	- Sinto que tenho de fazer força para a minha voz sair	**Sinto que tenho de fazer força para a minha voz sair**	- Sinto que faço força para a minha voz sair	Sinto que faço força para a minha voz sair
	- Sinto necessidade de fazer força para a minha voz sair		- Sinto que tenho que fazer força para a minha voz sair	
2. Minha voz é rouca	- A minha voz é rouca	**A minha voz é rouca**	- A minha voz é rouca	A minha voz é rouca
	- A minha voz é rouca		- A minha voz é rouca	

Os participantes apresentaram disfonia funcional (n=11; 36,67%), organofuncional (n=9; 30%) e orgânica (n=10; 33,33%), com diagnóstico médico otorrinolaringológico. A Tabela 1 mostra que nenhum participante respondeu não aplicável para os itens do instrumento. Para o item “sinto que tenho de fazer força para a minha voz sair”, 46,67% dos participantes responderam não e 53,33% responderam sim. Já para o item “a minha voz é rouca”, 20% dos participantes responderam não e 80% responderam sim.

**Tabela 1:** Proporção de respostas nos itens do instrumento

Variável e categorias	n	%	p-valor
Sinto que tenho de fazer força para a minha voz sair			
Não/Sim	30	100,00	1,000
Não aplicável	0	0,00	
A minha voz é rouca			
Não/Sim	30	100,00	1,000
Não aplicável	0	0,00	

Teste Binomial

**Legenda:** n=frequência absoluta; %=frequência relativa

## DISCUSSÃO

A adaptação transcultural visa a adequação dos itens de um instrumento para a sua aplicabilidade em uma população de outro idioma ou cultura. Ela permite que as possíveis diferenças socioculturais entre as culturas e os idiomas sejam resolvidas, não sendo uma mera tradução literal do instrumento original, possibilitando o seu uso em um formato adequado aos indivíduos da cultura-alvo^(10)^. Somente após a adaptação transcultural o instrumento pode ser utilizado em uma segunda língua e cultura.

A tradução do IRD-Br para o PE não apresentou divergências de conteúdo. Na chave de resposta não houve divergência ou necessidade de ajuste. Com relação ao título, houve concordância na tradução, porém, na retrotradução surgiram duas possibilidades: *“questionário para a triagem”* ou *“instrumento de rastreio”*. Optou-se por manter o termo instrumento pela conceituação e pelo formato de uso do IRD. O conceito “*questionário*” é mais limitado que o conceito de *“instrumento"*. Questionário é um instrumento de coleta de dados, que contém uma série ordenada de perguntas. Já instrumento, é o que será utilizado no desenvolvimento do estudo para a obtenção de dados^(11)^, podendo ter formas específicas de análise que respaldem uma tomada de decisão.

O IRD-Br não busca somente a obtenção de dados, mas também oferece uma fórmula para a tomada de decisão do clínico consoante a resposta obtida pelo indivíduo investigado. Entende-se que ele não seja somente um questionário para obtenção de informações isoladas.

Elegeu-se o conceito de "rastreio" em detrimento de "triagem", visto que rastreio envolve a detecção simples, econômica e rápida de uma provável doença ou lesão em qualquer indivíduo, seguido por um encaminhamento para diagnóstico de confirmação e tratamento. A triagem, por sua vez, identifica indivíduos afetados por uma doença ou distúrbio em uma população, a fim de direcioná-los para procedimentos diagnósticos mais completos^(7)^. Perante tais definições, o conceito de "rastreio" foi escolhido pelo comitê de especialistas, pois está de acordo com a proposta do IRD-Br, que é a de detetar de forma simples, econômica e rápida indivíduos disfônicos e encaminhá-los para diagnóstico de confirmação e tratamento.

Com relação aos itens, na etapa de consenso da tradução e da retrotradução utilizou-se como premissa a simplificação. A simplificação sintática é uma prática da psicolinguística apoiada em evidências de que determinadas características gramaticais podem impor maiores ou menores dificuldades para a compreensão de um texto^(12)^. Nesse sentido, a simplificação parte de diferentes estratégias destinadas a diminuir a complexidade estrutural do texto, sem modificar o conteúdo da informação original, ou seja, o seu significado, o que permite atingir de modo mais amplo, indivíduos de diversos níveis socioeconômicos e culturais. Com base nisso, o primeiro item apresentou divergência na tradução, sendo definida a tradução como: *“Sinto que tenho de fazer força para a minha voz sair”*. Houve divergência também na retrotradução, por isso na análise do comitê de especialistas ele foi modificado para *“Sinto que faço força para a minha voz sair”*, tornando o item mais simples e de clara interpretação. O segundo item não sofreu alteração em nenhuma das etapas, mantendo a mesma versão desde a etapa de tradução. Houve mudança em relação a versão em português brasileiro apenas pela inserção do artigo *“a”* desde a primeira etapa, para a sentença se tornar mais clara no PE.

O instrumento original não tem enunciado formal para introduzir as questões que o compõem. Porém, sentiu-se a necessidade de colocar um enunciado no instrumento, principalmente pela questão da aplicabilidade online, para tornar mais clara a intenção do instrumento, e trazer instruções de preenchimento para a população. Considerando os dois instrumentos (IDV-10; VoiSS) que têm por base a criação do instrumento em questão, optou-se por elaborar o enunciado baseando-se na versão em PE desses instrumentos. Foram elaboradas quatro opções de enunciado, dos quais os autores escolheram a opção: “Responda aos dois itens abaixo, considerando a sua voz atualmente”.

A opção “não aplicável” não foi assinalada pelos participantes no pré-teste. Os resultados obtidos nessa fase são considerados excelentes, pois os 30 participantes responderam afirmativamente ou negativamente aos dois itens estudados, ou seja, eles compreenderam os itens e responderam com opções da chave de resposta do próprio instrumento. Isso mostra que a adaptação do instrumento para o PE resultou em uma versão de fácil compreensão à população portuguesa.

A adaptação transcultural é a primeira etapa para a validação de instrumentos, pois todo o restante do processo é realizado a partir dela^(10)^. Com a conclusão do processo de adaptação transcultural para o PE, torna-se necessário o desenvolvimento de uma pesquisa de validação do IRD-PT, a fim de demonstrar as propriedades psicométricas de validade, confiabilidade e acurácia da versão portuguesa, e possibilitar o uso confiável em pesquisas e na prática clínica^(13)^.

## CONCLUSÃO

A tradução e adaptação do IRD-Br para a população portuguesa foi bem sucedida. A versão em português europeu do IRD-Br foi denominada Instrumento de Rastreio para a Disfonia em português europeu - IRD-PT (Anexo 1).

## Anexo 1. INSTRUMENTO DE RASTREIO PARA A DISFONIA - IRD^PT^

Responda aos dois itens abaixo, considerando a sua voz atualmente

**Table:**

1) Sinto que tenho de fazer força para a minha voz sair	( ) Sim ( ) Não
2) A minha voz é rouca	( ) Sim ( ) Não
